# The relation between apomictic seed production and morpho-physiological characteristics in a world collection of castor bean (*Ricinus communis* L.)

**DOI:** 10.1038/s41598-024-53700-1

**Published:** 2024-02-29

**Authors:** Maedeh Setayeshnasab, Mohammad R. Sabzalian, Mehdi Rahimmalek

**Affiliations:** 1 Department of Agronomy and Plant Breeding, College of Agriculture, Isfahan University of Technology, 84156-83111 Isfahan, Iran; 2 Department of Horticulture, College of Agriculture, Isfahan University of Technology, 84156-83111 Isfahan, Iran

**Keywords:** Plant sciences, Plant breeding, Plant reproduction

## Abstract

*Ricinus communis* is one of the most important oilseed plants with many medicinal and industrial applications. Variation in 30 genotypes of castor bean collected from different regions of the world was evaluated for two consecutive years and the difference in seed production with two different reproductive modes (including apomixis and open-pollination) was compared based on yield components, agronomic traits, and phytochemical properties. Results of data analysis demonstrated that castor bean has the ability for a wide range of apomixis for seed production and the highest percentages of apomixis ability in the first and second years were 86.3% and 92.31%, respectively. Apomixis ability had a high positive correlation with yield components, seed oil content, and the amount of leaf rutin. Two genotypes from Brazil and Syria revealed the highest phenolic content in the first and second years, respectively. In addition, the Afghanistan genotype in two modes of apomixis and open-pollination in the first year and the Syria and Yazd genotypes in apomixis and open-pollination modes, respectively, in the second year showed the highest content of seed fatty acids. It is possible to maintain superior genotypes of castor bean in terms of phytochemical traits, yield, and oil quality through apomixis reproduction.

## Introduction

Castor bean (*Ricinus communis* L., 2n = 2X = 20) is a monophyletic species belonging to the Euphorbiaceae family, which includes 280 genera and 8000 species. The origin of *R. communis* is Africa and then it was taken to India and China. Afterward, the plant spread around the world from temperate to tropical areas^1^. Castor bean is a dual-purpose medicinal and oil plant^2^. The medicinal properties of *R. communis* are due to the presence of some phytochemical compounds like flavonoids, phenolic acids, glycosides, alkaloids, steroids, and terpenoids. The antioxidant activity of phytochemical components of this plant is considered in the treatment of tumors and cancers. In addition, the plant is reported to possess other medicinal properties such as anti-diabetic, anti-microbial, anti-viral, anti-aging, anti-dermatophytic, anti-inflammatory, anti-nociceptive, and anti-hepatotoxic^3–6^.

Castor bean seed with 45–55% oil content is one of the most important oilseeds which contains a high percentage of ricinoleic acid. The special physicochemical properties of castor bean oil (including solubility in alcohol, high viscosity, and requiring low heat in the biodiesel production process) make it suitable for various industrial, pharmaceutical, and cosmetic uses. Industrial applications of castor bean oil include biodiesel production, waterproof coating, polymeric materials, lubricant, candle, brake fluid, shoe wax, and carbon paper. In addition, castor bean oil is highly valued in the treatment of diseases such as dry eye, meibomian gland dysfunction, wounds, constipation, and for cosmetic products such as shampoo, soap, and lotion^1,5,7^.

*R. communis* can grow in poor soils and stressful climates and is therefore considered as one of the agricultural solutions for areas with limited resources^8^. This plant is currently grown on a commercial scale in more than 30 countries, including India, Brazil, China, and Thailand^9^. According to FAO reports, the average global production, area under cultivation, and yield of castor bean seeds in 2021 are estimated to be 1,861,700 tons, 1,296,895 ha, and 1435.5 kg/ha, respectively. The leading producing countries in 2021 included India (1,647,000 tons), Mozambique (72,783 tons), Brazil (35,195 tons), China (21,000 tons), Thailand (12,000), Myanmar (11,696 tons), Ethiopia (11,000 tons), Vietnam (7000 tons), South Africa (6519 tons), and Paraguay (6000 tons).

Castor bean is a single-stemmed plant and its flowers are unisexual, producing male and female flowers on dichasial cymes. Male and female buds are different in terms of size, shape, and location on the inflorescence. The female buds are larger than the male buds, oval shape, and are located at the top of the inflorescence, while the male buds are round and are at the bottom of the inflorescence. As a result of this inflorescence structure, castor bean is reported to be more than 80% open-pollinating and its pollination is done by wind or insects. Also, before the male flowers open, the female flowers produce seeds, which also helps the open-pollination nature of the plant^1^. Castor bean plants can self-pollinate if isolated by distance or pocketing^10^. In addition to the reproductive modes mentioned, personal studies on this plant provided evidence of facultative apomixis. This mechanism of reproduction may affect many traits related to genetic diversity and breeding, population survival, and crop production, but no report on this mode of seed production in castor bean has been offered so far.

Sexual reproduction creates genetic diversity among plant species, which is necessary to improve crop quality in agriculture. On the other hand, sexual reproduction segregates advantageous traits in the next generations, which is a weakness of sexual reproduction. In some species, in addition to sexual reproduction, they can reproduce asexually, a process known as apomixis^11^. Apomixis is the mechanism of seed formation without fertilization and has been observed in more than 400 species of flowering plants (32 plant families). Events of sexual reproduction (meiosis and fertilization) do not happen in apomixis. In apomixis, the progression of meiosis is interrupted in the first or second cycle. As a result, oocyte fertilization does not occur in apomixis development, but the fertilization of polar cells causes endosperm formation^12^.

Most of the apomictic plants are facultative ones and can reproduce in both sexual and apomixis forms. Apomixis can be divided into two types, gametophytic and sporophytic. In sporophytic, the clonal embryo arises from a somatic cell in the tissue around the ovule. In this type of plants, sexual reproduction also takes place and inside the seed, there is a sexual embryo and one or more asexual embryos (polyembryony). Sporophytic apomixis is found in *Citrus* species, mango, and orchids. Gametophytic apomixis is known by two mechanisms: apospory and diplospory. In diplospory, the embryonic sac is made by mitosis or after disrupted meiosis by the megaspore mother cell. Diplospory has been reported in *Tripsacum*, *Eragrostis*, and *Taraxacum*. Also, in apospory, one or more ovule somatic cells are distinguished by a structure that is similar in shape and function to that of the megaspore. Apospory is the most common mechanism of apomixis in higher plants and is common in different genera including *Beta*, *Brachiaria*, *Cenchrus*, *Chloris*, *Eriochloa*, *Heteropogon*, *Hieracium*, *Hyparrhenia*, *Hypericum*, *Panicum*, *Paspalum*, *Pennisetum*, *Ranunculus*, *Sorghum*, *Themeda*, and *Urochloa*^13–16^.

Apomixis reproduction is not common in cultivated species and is commonly observed in wild plant species^16^. Although there is a prominent connection between polyploidy and gametophytic apomixis, the discovery of apomixis in diploids and the prevalence of diploidy in sporophytic apomictic plants rejects the hypothesis of the necessity of polyploidy in the development of apomixis^17,18^. Also, the evidence obtained from research on model plant species shows that apomixis is the result of epigenetic changes that occur in plants due to incorrect regulation of reproductive pathways and transcriptional modifications, resulting from the processes of polyploidization and distant hybridization^19^.

Studies on apomixis reproduction describe this process as a form of reoriented sexual reproduction. Apomixis emerges from the deregulated expression of sex-related genes as a result of asynchronous gene expression, gene duplication, and hybridization in sexual species and the evidence is studies conducted on *Tripsacum sp.* and *Boechera sp.*^20,21^.

The studies conducted on apomictic and sexual plants have shown that apomixis is a heritable dominant trait controlled by a single locus^22,23^. Apospory and diplospory in grasses are controlled by a single dominant locus^24^. Also, a simple dominant gene for apomixis has been reported in *Panicum maximum* and *Hieracium aurantiacum*^25,26^. However, few studies reported apomixis as an oligogenic trait controlled by a few genes^27^. In addition, other studies indicate an independent dominant control for different components of apomixis (apomeiosis, parthenogenesis, and endosperm development)^28^. In past studies, pleiotropy was reported for apomeiosis and parthenogenesis, but studies in the genera *Allium*, *Poa*, *Erigeron*, *Taraxacum*, *Hieracium*, *Hypericum*, and *Potentilla* showed independent control of these traits^29^. Furthermore, polygenic control on each apomixis component has been identified in *Poa pratensis*^30^.

Apomixis combines the benefits of seed propagation (high propagation rate, easy storage and planting, suitability for planting by machine, less seed use, and less disease spread) with vegetative cloning (preservation of genetic structure and fixation of superior genotypes after crossing). Apomixis has many benefits and applications such as propagation of superior genotypes including hybrid plants in the form of seeds, reduction of hybrid seed prices, farmers' self-sufficiency in seed production, no need for a male sterile system in hybrid production, production of new varieties with a single cross without creating homozygosity, and increasing survival of interspecific crosses^16^. In addition, after identifying the genes involved in asexual reproduction, apomixis and its benefits can be transmitted through gene transformation to other plants^31^.

As mentioned, no research has been reported yet on the ability of castor bean for the asexual mode of seed production by apomixis. Sexual reproduction changes excellent traits in later generations, while asexual reproduction by seed (apomixis) transmits superior traits (phytochemical properties and oil content) to the offspring without any change. In this research, seed production in castor bean was performed in two ways: apomixis and open-pollination. Additionally, yield components, agronomic traits, and phytochemical properties were measured to assess variations among different genotypes and compare seed production using the two reproductive modes. We hypothesized that apomixis ability in castor bean is connected to some morpho-physiological characteristics in a world collection of the plant (Table 1).

**Table 1:** Details of plant material used in this study (provided by the USA seed bank).

Number	Accession number	Country of origin	Accession designation
1	PI 27477201	Argentina 1	ARG1
2	PI 22105101	Argentina 2	ARG2
3	PI 27702001	Argentina 3	ARG3
4	PI 16316202	Brazil 1	BRA1
5	PI 20271101	Brazil 2	BRA2
6	PI 43959001	Brazil 3	BRA3
7	PI 64200003	USA 1	USA1
8	PI 63115601	USA 2	USA2
9	PI 20884101	Cuba 1	CUB1
10	PI 20962201	Cuba 2	CUB2
11	PI 18106301	India 1	IND1
12	PI 24894201	India 2	IND2
13	PI 26793701	India 3	IND3
14	PI 20183001	Madagascar	MDG
15	PI 21211501	Afghanistan	AFG
16	PI 27186101	Ecuador	ECU
17	PI 27477101	South Africa	ZAF
18	PI 20651501	Jamaica	JAM
19	PI 16544603	Mexico	MEX
20	PI 24031001	Benin	BEN
21	PI 24844101	Congo	COG
22	PI 25362102	Morocco	MAR
23	PI 63878801	Israel	IL
24	PI 16707202	Turkey	TUR
25	PI 25062201	Pakistan	PAK
26	PI 18191601	Syria	SYR
27	PI 25093801	Iran	IRN
28	PI 25765303	Russia	RUS
29	PI 25057501	Egypt	EGY
30	PI 32358901	Portugal	PRT

## Results

### Mean comparisons of yield components and agronomic traits

In the first year (Table S1), Yazd had the highest number of seeds per raceme in two modes of apomixis (114) and open-pollination (182). The highest amount of 100-seed weight was observed in Tehran in two modes of apomixis (47.48 g) and open-pollination (43.37 g). Also, the amount of 100-seed weight in most genotypes in apomixis reproduction was higher than open-pollination reproduction. Isfahan (25.31 g) and Tehran (51.41 g) had the highest seed weight per raceme in apomixis and open-pollination modes, respectively. Among all genotypes evaluated, the highest seed yield per plant in the two reproductive modes belonged to Iran (2813.98 and 5523.60 g in apomixis and open-pollination, respectively). The highest value for other traits including the number of racemes per plant, height, diameter and fresh, and dry weights belonged to Turkey (229), Madagascar (254.47 cm), Pakistan (5.74 cm), India 3 (3102.11 g), and India 3 (1217.41 g), respectively (Table S1).

In 2021, Yazd once again had the highest number of seeds per raceme in both apomixis and open-pollination modes, with counts of 109 and 163, respectively. The amount of 100-seed weight in the second year was also higher in the apomixis mode than the open-pollination mode and the highest amount belonged to Tehran in the apomixis mode (48.03 g) like the first year, and India 1 in the open-pollination mode (56.38 g). Yazd (24.11 g) and Tehran (46.10 g) had the highest seed weight per raceme in apomixis and open-pollination modes, respectively. Similar to the first year, Iran had the highest seed yield per plant in both reproductive modes (2555.02 and 4702.75 g in apomixis and open-pollination, respectively). Furthermore, the highest number of racemes per plant, height, diameter, fresh, and dry weights were observed in Turkey (218.67), India 3 (231.33 cm), Morocco (5.27 cm), India 3 (2820.28 g), and Iran (1106.34 g), respectively (Table S2).

According to the results obtained from the present study, the effect of year was significant in all agricultural traits and yield components except for seed number per raceme and seed weight per raceme in the apomixis mode (Table 2). Also, the interaction effect of genotype by year for the traits of 100-seed weight in the two modes of reproduction and seed yield per plant in the open-pollination mode was significant.

**Table 2:** Combined analysis of variance for yield components, agronomic and phytochemical traits in 33 genotypes of castor bean (two modes of reproduction, apomixis and open-pollination) in two consecutive years.

Source of Variation	DF	Mean square										
		No. seeds/raceme		100-seed weight		Seed weight/raceme		Seed yield/plant		Oil content		Apomixis ability
		Apo	Open	Apo	Open	Apo	Open	Apo	Open	Apo	Open	
Year	1	88.56^ns^	1282.88**	3.21**	13.76**	1.70^ns^	83.82**	95042**	1074035**	15.26**	15.35**	217.07^ns^
Block (year)	8	54.82	25.5	0.37	0.13	0.81	0.41	1446.98	2480.66	0.001	0.006	304.31
Genotype	32	2856.83**	6564.90**	229.93**	180.55**	129.32**	387.96**	883993**	2164124**	23.24**	15.62**	585.66**
Year* Genotype	32	10.50^ns^	23.00^ns^	4.34**	13.84**	0.68^ns^	1.31^ns^	5415.69^ns^	16773*	0.003^ns^	0.002^ns^	40.50^ns^
Error	16	45.94	17.65	0.062	0.09	0.87	1.38	4617.35	7652.87	0.01	0.004	77.24
CV (%)	–	12.76	4.38	0.85	1.13	6.79	4.91	7.39	5.73	0.26	0.13	16.40

S. V	DF	Mean square										
		No. racemes/plant	Height	Diameter	Fresh weight	Dry weight	Chl a	Chl b	Carotenoid	Phenolic content	Flavonoid content	IC50
Year	1	319.35**	5703.78**	2.53**	0.58**	0.09**	0.01**	0.002**	0.0002^ns^	618.44^ns^	964.31**	0.71**
Block (year)	8	5.18	117.75	0.01	0.02	0.009	0.0006	0.0003	0.0002	244.93	87.01	0.02
Genotype	32	4875.89**	1123.12**	0.87**	0.54**	0.07**	0.07**	0.007**	0.003**	9834.08**	654.05**	0.59**
Year*Genotype	32	4.17^ns^	23.22^ns^	0.004^ns^	0.004^ns^	0.001^ns^	0.04^**^	0.004**	0.002**	8179.41**	443.45**	0.53**
Error	16	20.13	77.95	0.04	0.04	0.009	0.0004	0.0001	0.0002	217.22	62.30	0.02
CV (%)	–	5.97	4.64	4.32	9.84	12.61	1.55	3.32	3.94	4.64	4.75	5.32

*, **, and NS indicate significance at the probability level of 5%, 1% and non-significant, respectively.

### Apomixis ability

In the present study, an attempt had been made to investigate the apomixis ability of reproduction in the studied genotypes. Based on the results from this study (Tables S1 and S2), apomixis reproduction ability varied by the genotype and ranged from 20.09% to 86.3% in 2020 and from 27.42% to 92.31% in 2021. The highest percentage of apomixis ability in both years was related to the Brazil 1 genotype. In addition, the effect of year and interaction effect of genotype by year was not significant (Table 2).

### Oil content of seeds

In this study, seed oil was extracted from apomictic and open-pollinated seeds of each genotype and the percentage of seed oil was calculated. In 2020, the maximum oil content was observed in the Afghanistan genotype with 51.25% in apomixis mode and 50.42% in open-pollination mode (Table S1). In 2021, the maximum oil content was obtained in the Syria genotype with 50.2% in apomixis mode and Yazd with 49.4% in open-pollination mode (Table S2).

The observations obtained from the present study indicate that the effect of year was significant in oil content in the two reproductive modes and the interaction effect of genotype by year was not significant in the two reproductive modes (Table 2).

### Phytochemical traits

According to phytochemical data from the first year (Table S1), the highest amounts of chlorophyll *a*, chlorophyll *b*, and carotenoid were present in the genotypes of Afghanistan (1.63 mg/g Fw), Mexico (0.47 mg/g Fw), and Madagascar (0.39 mg/g Fw), respectively. In addition, the highest amounts of TPC (589.22 mg gallic acid per gram of dry weight) and antioxidant activity (low IC_50_) (55.65 µg/ml) were observed in Brazil 2 and Cuba 2 had the highest amount of TFC (220.5 mg quercetin per gram of dry weight). Furthermore, in the second year (Table S2), the highest levels of chlorophyll *a*, chlorophyll *b*, and carotenoid were observed in Iran (1.62 mg/g Fw, 0.46 mg/g Fw, and 0.39 mg/g Fw, respectively). Syria also had the highest level of TPC (657.89 mg GAE/g of dry weight) and antioxidant activity (47.72 µg/ml) and India 2 had the highest amount of TFC (218.71 mg QE/g of dry weight).

The results of the examined traits in this study showed that the effect of year was significant for all phytochemical traits except for carotenoid and phenolic content, although the interaction effect of genotype by year was significant for all phytochemical traits (Table 2).

### Cluster analysis

**Figure 1:**
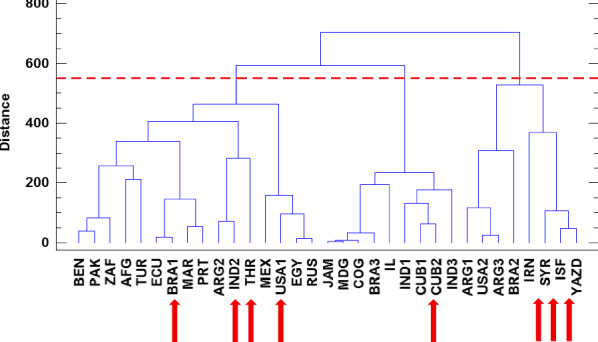
Cluster analysis for genotypes of castor bean based on yield components, oil content, agronomic and phytochemical traits in 2020. Red arrows show selected genotypes for GC and HPLC analyses.

**Figure 2:**
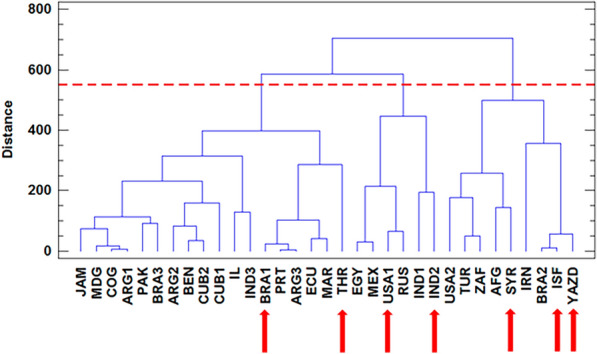
Cluster analysis for genotypes of castor bean based on yield components, oil content, agronomic and phytochemical traits in 2021. Red arrows show selected genotypes for GC and HPLC analyses.

The cluster analysis of the castor bean genotypes based on yield components, oil content, and agronomic and phytochemical data in the two reproductive modes was performed for each of the two years of study. Figures 1 and 2 present the corresponding dendrograms using Ward’s method and grouped the genotypes based on the CCC (Cubic Clustering Criterion) plot of the statistical software. Genotypes were grouped into three clusters in each year. The first cluster in 2020 included 16 genotypes, all with the highest 100-seed weight in apomixis and open-pollination reproductions and IC_50_. The second cluster with 9 genotypes was characterized by the highest amount of carotenoid and the third cluster with 8 genotypes was characterized by the highest number of seeds per raceme, seed weight per raceme, and seed yield per raceme in two modes of apomixis and open-pollination, apomixis ability, and leaf phenolic content.

In 2021, the first cluster included 18 genotypes, all with the highest 100-seed weight in the mode of apomixis, diameter, and IC_50_. The second cluster included 6 genotypes, all with the highest 100-seed weight in open-pollination mode and the third cluster also with 9 genotypes was characterized by the highest number of seeds per raceme, seed yield per raceme, and oil content in two modes of apomixis and open-pollination, seed weight per raceme in open-pollination mode, apomixis ability, and the number of racemes per plant.

Based on the results obtained, it can be concluded that the genotypes placed in the third cluster in both years are superior in traits of apomixis ability and yield components. Therefore, the same genotypes in the third cluster in both years, i.e., USA 2, Brazil 2, Iran, Syria, Isfahan, and Yazd had the highest apomixis ability among the studied genotypes. As shown, the castor bean collection used in this study was not grouped based on geographical distribution.

Based on the cluster analysis, Syria, USA 1, Brazil 1, Cuba 2, India 2, Tehran, Isfahan, and Yazd were selected from both years for future analyses (Figs. 1 and 2).

### GC analysis

The fatty acid profile was determined to compare the composition and percentage of fatty acids in selected apomictic and open-pollinated seeds (including control genotypes and genotypes selected based on the cluster analysis) in two years (Table 3). The highest amount of palmitic acid in the first year was observed in two modes of reproduction in Cuba 2 (apomixis, 1.97% and open-pollination, 2.24%), and in the second year in apomictic and open-pollinated modes in Yazd (1.93%) and Cuba 2 (2.17%), respectively. Cuba 2 had the highest content of stearic acid in both years and both reproductive modes (2.25% and 2.59% in the first year and 2.06% and 2.41% in the second year in apomictic and open-pollinated seeds, respectively). The highest percentages of oleic and linoleic acids were found in apomictic seeds of Brazil 1 in both years (7.53% oleic acid and 9.4% linoleic acid in 2020 and 7.36% oleic acid and 9.17% linoleic acid in 2021) and in open-pollinated seeds in Cuba 2 (7.25% oleic acid and 8.93% linoleic acid in 2020 and 6.96% oleic acid and 8.61% linoleic acid in 2021). In the first year, Cuba 2 exhibited the highest level of linolenic acid in both apomixis (0.85%) and open-pollination (1%). In the second year, Isfahan (0.81%) and Cuba 2 (0.75%) had the highest linolenic acid content in apomixis and open-pollination, respectively. USA 1 contained the highest percentage of eicosenoic acid in both years and both reproductive modes (0.7% and 0.64% in the first year and 0.65% and 0.6% in the second year in apomictic and open-pollinated seeds, respectively).

**Table 3:** Fatty acid profile (%) of selected genotypes of castor bean (apomictic and open-pollinated seeds) in two consecutive years.

Fatty acid	Year	Genotype	USA 1		IND 2		BRA 1		CUB 2		SYR		THR		ISF		YAZD	
		Type	Apomict	Open	Apomict	Open	Apomict	Open	Apomict	Open	Apomict	Open	Apomict	Open	Apomict	Open	Apomict	Open
Palmitic acid (C16:0)	2020		1.68	1.64	1.53	1.60	1.96	1.45	1.97	2.24	1.63	1.69	1.58	1.33	1.81	1.60	1.87	1.51
	2021		1.57	1.52	1.67	1.72	1.89	1.37	1.91	2.17	1.75	1.80	1.50	1.24	1.91	1.68	1.93	1.56
Stearic acid (C18:0)	2020		1.79	1.83	1.44	1.52	2.16	1.38	2.25	2.59	1.61	1.74	1.97	1.83	1.90	1.69	1.94	1.60
	2021		1.70	1.75	1.61	1.70	1.93	1.16	2.06	2.41	1.71	1.85	1.87	1.74	1.98	1.78	2.00	1.68
Oleic acid (C18:1)	2020		6.32	6.51	4.59	5.42	7.53	5.62	6.63	7.25	5.60	5.02	6.33	5.74	5.52	5.04	5.97	4.67
	2021		6.13	6.31	4.81	5.63	7.36	5.44	6.36	6.96	5.76	5.17	6.21	5.61	5.62	5.13	6.09	4.78
Linoleic acid (C18:2)	2020		7.97	7.62	6.54	6.84	9.40	6.86	8.55	8.93	6.26	6.79	7.49	6.51	7.94	6.48	7.46	6.38
	2021		7.80	7.46	6.66	6.97	9.17	6.64	8.22	8.61	6.38	6.92	7.39	6.42	8.02	6.57	7.59	6.52
Linolenic acid (C18:3)	2020		0.73	0.64	0.62	0.64	0.81	0.54	0.85	1.00	0.65	0.63	0.59	0.53	0.75	0.69	0.74	0.55
	2021		0.69	0.59	0.68	0.71	0.72	0.44	0.61	0.75	0.77	0.74	0.58	0.51	0.81	0.74	0.79	0.59
Eicosenoic acid (C20:1)	2020		0.70	0.64	0.36	0.49	0.65	0.48	0.44	0.49	0.38	0.35	0.55	0.54	0.29	0.31	0.40	0.33
	2021		0.65	0.60	0.39	0.53	0.59	0.43	0.38	0.44	0.41	0.39	0.51	0.51	0.32	0.35	0.42	0.36
Ricinoleic acid (C18:1 OH)	2020		80.81	81.12	84.93	83.49	77.51	83.68	79.31	77.51	83.87	83.77	81.49	83.52	81.79	84.20	81.62	84.97
	2021		81.45	81.77	84.18	82.74	78.36	84.53	80.46	78.66	83.22	83.12	81.94	83.97	81.34	83.75	81.17	84.52

The highest content of ricinoleic acid (the major component present in castor bean oil) in both years in apomixis was related to India 2 (84.93% in 2020 and 84.18% in 2021) and in open-pollinated ones was related to Yazd (84.97%) and Brazil 1 (84.53%), in the first and second years, respectively.

### HPLC analysis

Flavonoid and phenolic compounds of selected genotypes (including control genotypes and genotypes selected based on the cluster analysis) of castor bean were revealed by high performance liquid chromatography (HPLC) at 270 and 330 nm. HPLC results of eight flavonoid and phenolic compounds are shown in Table 4 as mg per 100 g dry weight of the samples. According to the results of extract analysis of leaves, syringic acid was the most abundant phenolic compound in most genotypes. The highest amount of gallic acid was present in Yazd (40.46 mg/100 g) in the first year and in Syria (64.26 mg/100 g) in the second year. Yazd (266.24 mg/100 g) and Cuba 2 (267.82 mg/100 g) had the highest amount of hydroxybenzoic acid in 2020 and 2021, respectively. The highest content of vanillic acid was observed in USA 1 in both years (30.35 mg/100 g in the first year and 58.19 mg/100 g in the second year). Isfahan (270.42 mg/100 g in 2020 and 366.07 mg/100 g in 2021) and India 2 (267.63 mg/100 g in 2020 and 251.64 mg/100 g in 2021) had the highest amounts of syringic acid and ellagic acid, respectively, in both years. Also, the highest contents of *P*-coumaric acid, rutin, and ferulic acid were related to Syria (152.94 mg/100 g), Tehran (120.37 mg/100 g), and USA 1 (188.22 mg/100 g) in 2020 and India 2 (150.22 mg/100 g), Yazd (145.25 mg/100 g), and brazil 1 (175.66 mg/100 g) in 2021, respectively.

**Table 4:** Leaf phenolic and flavonoid compounds (mg/100 g dry weight) identified in selected genotypes of castor bean in two consecutive years.

Compound name	RT (min)	Genotype	USA 1		IND 2		BRA 1		CUB 2		SYR		THR		ISF		Yazd	
		Year	2020	2021	2020	2021	2020	2021	2020	2021	2020	2021	2020	2021	2020	2021	2020	2021
Gallic acid	4.93 ± 0.2		32.81	29.37	19.09	29.67	29.75	18.56	25.92	44.16	39.74	64.26	31.89	30.61	36.32	35.43	40.46	16.85
Hydroxybenzoic acid	13.81 ± 0.4		149.52	113.14	152.55	101.22	87.23	131.06	103.19	267.82	156.23	85.94	179.14	102.81	156.35	142.74	266.24	197.17
Vanillic acid	14.82 ± 0.7		30.35	58.19	26.13	20.24	29.41	43.60	24.99	22.13	24.7	22.61	18.65	14.88	25.70	24.43	29.75	28.51
Syringic acid	16.18 ± 0.3		196.54	105.85	190.34	202.74	204.51	241.27	225.05	109.65	129.92	102.93	114.32	226.93	270.42	366.07	191.60	266.48
Ellagic acid	18.13 ± 0.5		56.80	112.69	267.63	251.64	123.88	80.42	128.93	130.28	155.61	109.53	94.90	123.19	136.50	78.65	154.60	91.68
*P*-coumaric acid	26.79 ± 0.9		98.08	96.61	126.75	150.22	98.88	137.81	107.17	103.19	152.94	112.05	61.79	73.46	132.45	99.83	116.75	76.35
Rutin	27.68 ± 0.7		96.87	91.26	73.63	106.19	75.23	86.31	67.83	59.30	78.80	102.36	120.37	92.47	95.90	83.10	90.44	145.25
Ferulic acid	28.74 ± 0.2		188.22	123.23	132.03	159.81	127.88	175.66	167.78	119.96	116.81	125.61	110.16	134.29	133.01	112.00	127.75	84.06

The data were sorted based on the retention time of components.

### Relation between apomixis ability and different traits

**Figure 3:**
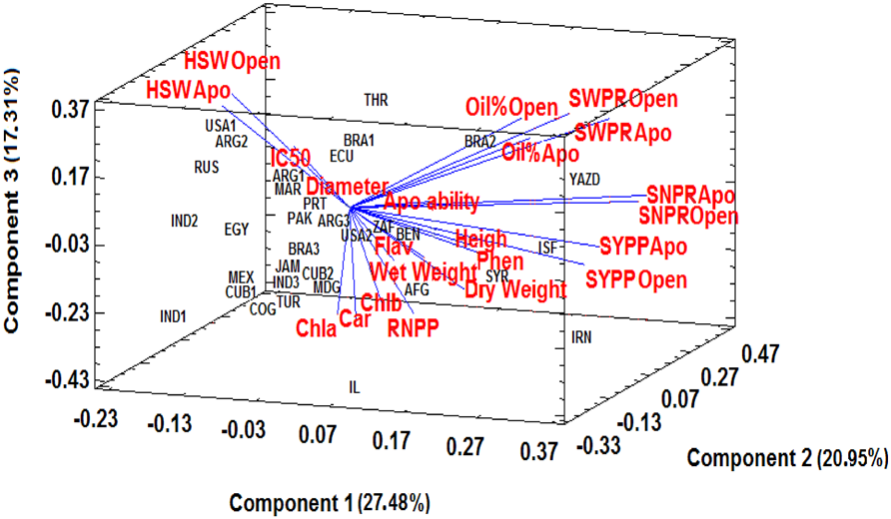
3D biplot of genotypes (33 genotypes of castor bean) based on yield components, oil content, agronomic and phytochemical traits in two modes of reproduction, apomixis and open-pollination in 2020, Apo: apomixis, Open: open -pollination, SNPR: seed number per raceme, HSW: 100-seed weight, SWPR: seed weight per raceme, SYPP: seed yield per plant, Apo ability: apomixis ability percentage, RNPP: number of racemes per plant, Wet Weight: fresh weight, Chla: chlorophyll *a*, Chlb: chlorophyll *b*, Car: carotenoid, Phen: phenolic content, Flav: flavonoid content.

**Figure 4:**
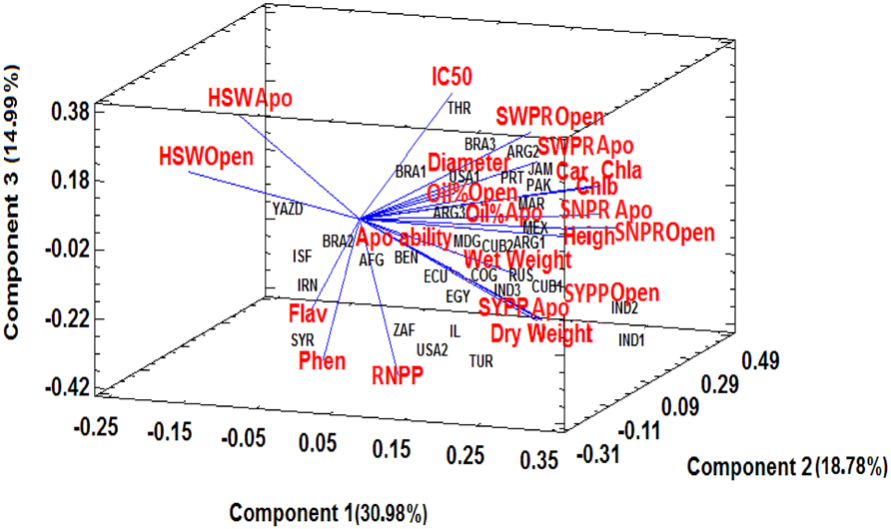
3D biplot of genotypes (33 genotypes of castor bean) based on yield components, oil content, agronomic and phytochemical traits in two modes of reproduction, apomixis and open-pollination in 2021, Apo: apomixis, Open: open -pollination, SNPR: seed number per raceme, HSW: 100-seed weight, SWPR: seed weight per raceme, SYPP: seed yield per plant, Apo ability: apomixis ability percentage, RNPP: number of racemes per plant, Wet Weight: fresh weight, Chla: chlorophyll *a*, Chlb: chlorophyll *b*, Car: carotenoid, Phen: phenolic content, Flav: flavonoid content.

The principal component analysis (PCA) was used to investigate the relationship between the apomixis ability of all genotypes with yield components, oil content, and agronomic and phytochemical traits in each year. The 3D components explained 27.48%, 20.95%, and 17.31% of the total variance in the first year (Fig. 3) and 30.98%, 18.78%, and 14.99% in the second year (Fig. 4). According to the results obtained from two years, the percentage of apomixis ability had a positive correlation with yield components including the number of seeds per raceme, seed weight per raceme, seed yield per plant, and seed oil content. In addition to the mentioned traits, the ability of castor bean apomixis is also positively correlated with the number of racemes per plant, plant height, stem diameter, fresh and dry weights, and leaf phenolic content.

**Figure 5:**
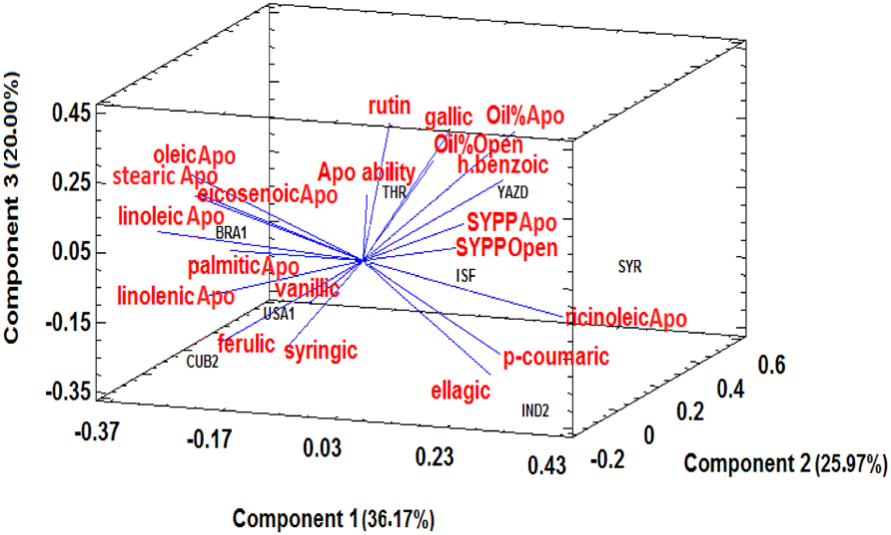
3D biplot of selected genotypes (8 genotypes of castor bean) based on the profile of seed fatty acids and leaf phenolic compounds in 2020, Apo: apomixis, Open: open -pollination, SYPP: seed yield per plant, Apo ability: apomixis ability percentage.

**Figure 6:**
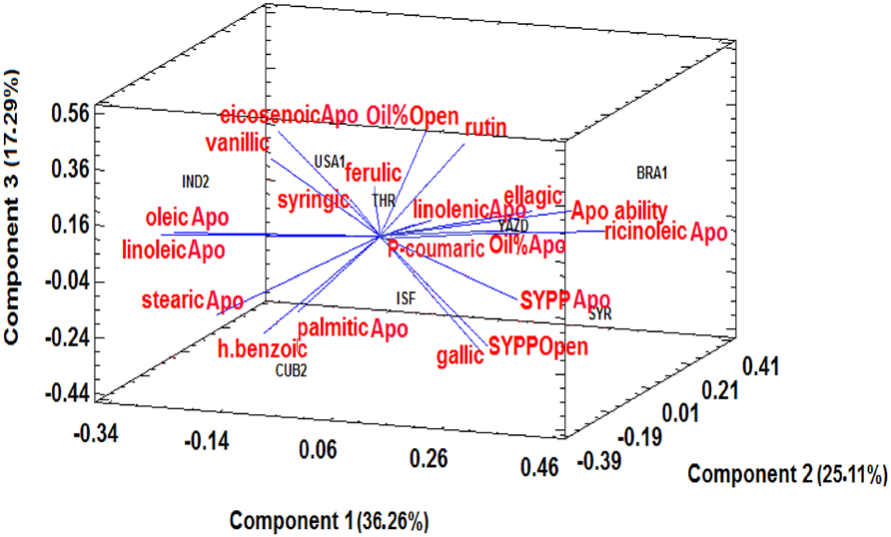
3D biplot of selected genotypes (8 genotypes of castor bean) based on the profile of seed fatty acids and leaf phenolic compounds in 2021, Apo: apomixis, Open: open -pollination, SYPP: seed yield per plant, Apo ability: apomixis ability percentage.

The PCA was also used to investigate the relationship between the apomixis ability of selected genotypes (including control genotypes and genotypes selected based on the cluster analysis) with the profile of seed fatty acids and leaf phenolic compounds in each year. The 3D components explained 36.17%, 25.97%, and 20.0% of the total variance in 2020 (Fig. 5) and 36.26%, 25.11%, and 17.29% in 2021 (Fig. 6). The results of this analysis revealed a strong positive correlation between the percentage of apomixis ability in selected genotypes and the levels of leaf rutin.

## Discussion

The results of this study on agronomic traits showed a higher variation compared with a study in which the agricultural characteristics of 68 genotypes of castor bean were investigated and the mean value of plant height, total number of racemes, 100-seed weight, and seed yield per plant were 69.61–203 cm, 5.38–14.4, 21.73–38.8 g, and 47.1–125.5 g, respectively^32^. Also, in another study, 82 germplasm lines were evaluated and plant height, 100-seed weight, and total yield per plant were reported 13.5–124 cm, 22.3–56.5 g, and 15–294.2 g, respectively^33^ which indicates a lower range of trait values compared to the results of the present study. This shows the broad-based variation of traits in a diverse panel of castor bean genotypes collected from around the world and used for generalization in this study.

In the study on phytochemical traits, there was a good accordance between the results of this study and those reported in the literature. In the study conducted by Wafa et al. on the leaves of five Tunisian castor bean populations, the ranges of TPC and TFC were reported as 174.25–623.7 mg GAE/g of dry matter and 99.6–213 mg QE/g of dry matter, respectively^34^. Also in another study, the ranges of TPC and TFC from leaves of two accessions of Mexican *Ricinus communis* were determined as 174.25–623.70 (mg GAE/g DW) and 201.75–234.92 (mg rutin equivalents/g DW)^35^.

Also, the results obtained from the oil content in this study were in accordance with the results of Rukhsar et al., who reported 46.75% to 51.75% of oil content in 15 castor bean genotypes^9^. In addition, oil content varied in different studies and was 44.5–54.2% in 19 genotypes^36^, 40.18–56.22% in 7 genotypes^37^, and 37–63% in 63 castor bean accessions^38^. In the studies conducted on *Jatropha curcas*, the oil content was reported 29.26% and 27.21% in 17 accessions^39^ and 34.4% and 35.9% in 33 accessions^40^ in apomictic and open-pollinated seeds, respectively.

According to recent reports, several factors such as the area of production, the type of cultivars, the geographical origin of the crop, the oil extraction process, the harvest period, and storage time affect fatty acid composition^41–43^. So far, there has been no report on the comparative study of the fatty acid profile in the two reproduction modes of apomixis and open-pollination. However, the present study showed that in addition to the factors mentioned, the mode of reproduction also affects the composition and percentage of fatty acids as apomixis may have a positive effect on oil content and a negative effect on ricinoleic acid content in most castor bean genotypes.

The results of HPLC analysis were in agreement with literature reports that conducted the isolation of phenolic compounds from the castor bean leaves. The phenolic compounds were gallic acid, vanillic acid, *p*-coumaric acid, rutin, and ellagic acid^34,35,44^. The present study revealed that there are other phenolic compounds such as Hydroxybenzoic acid, syringic acid, and ferulic acid which were not reported in the recent studies. Past studies have also shown that the amount of phenolic compounds in plants can be affected by several factors such as genetic diversity, climatic conditions, cultivation area, developmental stage, extraction method, and the standard of calibration^45^.

Among the literature, apomixis frequency varies in different studies and was reported at 25–96% in *Poa pratensis*^46^ and at 10–15% in diploid accessions and 80–95% in tetraploid accessions of *Brachiaria decumbens*^47^. In another study conducted on *Jatropha curcas*, the highest frequency of apomixis among 33 accessions, F_1_s created from the hybridization of two parents (non-toxic high-yielding genotype of Mexican origin × Indian toxic genotype with synchronous maturity) and F_1_A_1_ progeny (seeds obtained from the F_1_ hybrid through apomixis) were reported 37.5%, 87.9%, and 88.3%, respectively^40^. Considering that the apomixis reproduction in *Ricinus communis* is being reported for the first time in this study, there is a possibility that the seed production through self-fertilization in castor bean and inbreeding depression reported in the literature^10,48–50^ has been under the confounding effect of apomictic seed production.

According to the results obtained from two years of study on castor bean genotypes and analysis by the multivariate method, the percentage of apomixis ability may be positively correlated with yield components and some morpho-physiological characteristics, including leaf phenolic content. Particularly as mentioned, the results of this study showed that the percentage of apomixis ability had a high positive correlation with the amount of leaf rutin. These results were in line with the findings of Asker and Frost (1970), who revealed that numerous phenolic compounds were present in apomictic *Potentilla* species^51^. Similarly, Tateoka (1981) reported the presence of rutin in apomictic plants of *Calamagrostis sachalinensi*s and stated that a survey for the higher rutin content is useful in identifying apomictic plants. In addition, the study reported that while rutin was not detected in non-apomictic plants of *C. langsdorffi* at all, isoquercitrin (quercetin 3-glucoside) was recognized, which is a direct precursor of rutin^52^. This shows that rutin may somehow ameliorate apomictic reproduction.

Several studies have shown the stimulating effect of rutin, also called vitamin P, on many physiological and pharmacological activities including cell proliferation and anti-ROS activities^53^. It seems that during meiosis in sexual reproduction, the megaspore mother cell is enclosed by callose as a response to reactive oxygen species (ROS) and the proliferation of the female gametes in the absence of fertilization is also repressed by the activity of the MEA-FIE-PRC2 (for Medea (MEA), fertilization-independent endosperm (FIE), and polycomb repressive complex 2 (PRC2)) complex in the central cell proliferation and a repressive chromatin state in the egg cell. In contrast, in apomicts, quenching of ROS, the proliferation of the female gametes, and autonomous endosperm development occurred^54^ which are all probably affected by rutin content.

## Conclusion

The ability to reproduce sexually and by apomixis at the same time (facultative apomixis), allows the plant to use the advantages of two modes of reproduction. This study proves the ability of *Ricinus communis* to perform apomixis in addition to sexual reproduction. In the present study, we measured yield components and other agronomic and phytochemical traits in a diversity panel of 30 castor bean genotypes to evaluate variation among genotypes and compare two reproductive modes (apomixis vs. open-pollination) in two consecutive years. According to the results obtained, apomixis vs. open-pollination had a significant effect on yield components, oil content, and fatty acid profile in castor bean. The number of seeds per raceme, seed weight per raceme, and seed yield per plant were higher in open-pollination compared to apomixis. In contrast, in most genotypes, 100-seed weight and oil content were higher in apomixis reproduction. It was found that the ability of apomixis had positive correlations with yield components and some other agronomic traits (including the number of racemes per plant, number of seeds per raceme, seed weight per raceme, plant height, stem diameter, fresh and dry weights, and seed yield per plant), seed oil content, and phytochemical traits of phenolic and rutin contents. The ability of seed production by apomixis in castor bean may provide a way to maintain superior genotypes.

## Material and methods

### Plant material

Castor bean seeds used in this study including 30 genotypes from around the world were obtained from the American Seed Bank of the United States (Table 1). Three genotypes from Iran including Tehran, Isfahan, and Yazd were also evaluated as the control group in an augmented design.

### Procedure and experimental design

This research was conducted in two years (May to November in 2020 and 2021) at the research farm of Isfahan University of Technology, located in Isfahan, Iran (32° 30′ N, 51° 20′ E). In this region, the mean annual temperature and precipitation were 14.5 °C and 120 mm, respectively. The soil was calcareous (390 g/kg Ca‐carbonate equivalent, 5 g/kg organic C and 0.8 g/kg total N) with pH = 8. The soil was non‐saline with a soil bulk density of 1.04 g/cm3. Genetic material prepared from the USA seed bank (30 genotypes) and Iran (3 genotypes) was grown in the form of an augmented design. In this study, genetic populations were randomly cultured in five incomplete blocks with nine plots in each block. Each plot contained 5–10 plants (5 m long, with 50 cm spacing between the plants) and the planting depth was 5 cm. The distance between the blocks was one meter, and plots (genotypes) were also placed in each block at a distance of one meter.

### Measurement of yield components and agronomic traits

In each year of castor bean cultivation, seed production was done in two ways: apomixis and open-pollination. To produce seeds in apomixis mode, every single plant in rows was emasculated and bagged. The covered inflorescences were checked every three days (until the maturity of the female flower) for male buds. Non-pocketed plants also produced open-pollinated seeds. Afterward, yield components and agronomic traits including the number of racemes per plant, seed number per raceme, 100-seed weight, seed weight per raceme, seed yield per plant, apomixis ability percentage, plant fresh and dry weights, plant height, and stem diameter were measured to evaluate different genotypes and two types of seeds taken from the plants. To obtain dry weight, the samples were placed in an oven at 70° C for 7 days (as long as there was no moisture in the sample). The percentage of apomixis ability was calculated according to the following formula:

$$\mathrm{Apomixis \,ability \,percentage }=\frac{\mathrm{number \,of \,fruits \, (seeds) \,formed \,in \,apomixis \,mode}}{\mathrm{number \,of \,pistillate \,flowers \,covered}}\times 100$$

Also, the standard deviation (S$\overline{{\text{d}} }$) for the traits in the augmented design was calculated according to Federer’s formula^55^:

$${\text{S}}\overline{{\text{d}} }=\sqrt{\frac{\mathrm{MSE }(2{\text{C}}+1)}{{\text{C}}}}$$

The factor C in Federer’s formula represents the number of control genotypes in the augmented design.

The least significant difference (LSD) was then calculated for mean comparisons according to Federer’s formula^55^:

$$\mathrm{LSD }={{\text{t}}}_{\frac{\mathrm{\alpha }}{2},{\text{dfe}}}\times {\text{s}}\overline{{\text{d}} }$$

The degree of freedom (dfe) belongs to the variance analysis of control genotypes and α is equal to 0.05.

### Oil extraction

The percentage of seed oil was measured by the soxhlet extraction method (for seeds from two modes of reproduction). For this purpose, five grams of castor bean seeds were ground (1–2 mm particles) and wrapped in a filter paper. Then the samples were transferred to a soxhlet extractor and 250 ml of normal hexane (n-hexane) as the solvent was added. After 8 h and complete evaporation of the solvent, the oil content (%) was calculated based on the weight of the sample used.

### Fatty acid composition

Gas chromatography (GC) analysis was performed on selected genotypes (including control genotypes and genotypes selected based on the cluster analysis) to determine the fatty acid profile^56^. Accordingly, after dissolving 50 μl of the oil samples in 200 μl of hexane, 100 μl of methanolic KOH was added and the resulting mixture was incubated at room temperature for 5 min. Eventually, 40 mg of sodium bisulfate was added and the supernatant was collected. The GC analysis of the FAMEs was done on an Agilent 7890A gas chromatograph equipped with a flame ionization detector (FID). The column used in this experiment was a DB-WAX fused polyethylene glycol column (60 m, 0.25 mm inner diameter, 0.25 μm film thickness). The temperature program included holding the temperature at 170 degrees for five minutes, and then increasing it to 220 degrees at a rate of one degree per minute. The temperature used for the injector and the detector was 220 degrees. The carrier gas was Ultra-high-purity nitrogen at a flow rate of 1 ml/min. The quantified data were obtained from GC-FID area percentages without the use of correction factors.

### Sample preparation for the measurement of phytochemical traits

To measure the amount of chlorophyll, mature leaves were collected from different parts of the plant and stored at − 20 °C. Also, to measure other phytochemicals, mature leaves were collected from different parts of the plant and air-dried at room temperature. The dried leaves were completely powdered and 0.25 g of powders were weighed. To extract phenolic content, 25 ml of 80% methanol was added and placed on a shaker at 150 rpm for 24 h. Finally, the extracts were filtered 2–3 times with a filter paper.

### Determination of chlorophyll and carotenoid content

The amount of 0.2 g of leaves was powdered with liquid nitrogen. Then, leaf samples were ground by adding 80% acetone and reaching the volume to 10 ml with 80% acetone. The extracts were centrifuged for 10 min at 3000 rpm and the absorbance was measured at 663, 646, and 470 nm against a blank^57^.

$${\text{C}}_{{\text{a}}} = 12.21\;{\text{A}}_{663} {-}2.81\;{\text{A}}_{646}$$

$${\text{C}}_{{\text{b}}} = 20.13\;{\text{A}}_{646} {-}5.03\;{\text{A}}_{663}$$

$${\text{C}}_{{{\text{x}} + {\text{c}}}} = \left[ {1000\;{\text{A}}_{470} {-}3.27\;{\text{C}}_{{\text{a}}} {-}104\;{\text{C}}_{{\text{b}}} } \right]/227$$

Where C_a_, C_b_, and C_x+c_ stand for chlorophyll *a*, chlorophyll *b*, and carotenoid contents, respectively. Chlorophyll and carotenoid contents in solution (mg/L) were multiplied by the solution volume (L) and divided by the leaf weight (g). As a result, the obtained values were expressed as mg per g of leaf fresh weight.

### Determination of total phenolics

The total phenolic content (TPC) was measured using the Folin-Ciocalteu method with some modification^58^. At first, 125 μl of each extract was added to 375 μl of deionized water. Then 2.5 mL of 10% Folin-Ciocalteu’s reagent in a dark place was added. After 5 min, 2 ml of 7.5% sodium carbonate was added and placed at 45° C for 15 min. The absorbance was read at 765 nm against a blank. The calibration curve was drawn for different gallic acid dilutions and the total phenolic content of leaves was expressed as gallic acid equivalent (mg gallic acid per gram of dry weight).

### Determination of total flavonoid

The total flavonoid content (TFC) was determined by aluminum chloride colorimetric assay^59^. The amount of 1 ml of each extract was added to 4 ml of deionized water. Then 300 μl of 5% NaNO_2_ was added and after 5 min in a dark place, 300 μl of 10% AlCl_3_ was added. After 6 min, 2 ml of 1 *M* NaOH was added and each sample was made up to 10 mL with deionized water. The samples were mixed and the absorbance was measured at 510 nm against a blank. For the calibration curve, different quercetin dilutions were used and the total flavonoid content of leaves was expressed as quercetin equivalent (mg quercetin per gram of dry weight).

### Determination of antioxidant capacity

The antioxidant capacity of leaf extracts was evaluated using the DPPH (1, 1- Diphenyl- 2- Picryl Hydrazyl) radical scavenging activity method developed by Brand-Williams et al. (1995) with some modification^60^. Initially, different concentrations of each leaf extract (dilutions of 50, 100, 300, and 500 ppm) were made in methanol (80%) and 100 μl of each sample was poured into a tube. Different concentrations of butylated hydroxytoluene (BHT) were also used as the standard antioxidant. An aliquot of 5 ml of 0.1 m*M* methanolic solution of DPPH was added to the samples and placed in the dark for 30 min at 37° C. For the control, 100 μl of 80% methanol was mixed with 5 ml of 0.1 m*M* DPPH solution. The absorbance was measured at 517 nm against a blank. Radical scavenging activity was calculated by the following formula:

$$\mathrm{\% \,Radical \,scavenging \,activity}=\frac{\mathrm{Absorbance \,of \,control}-\mathrm{Absorbance \,of \,sample}}{\mathrm{Absorbance \,of \,control}}\times 100$$

For each sample, the IC_50_ value was obtained using a graph of concentrations and radical scavenging activities. The IC_50_ value is the concentration of a sample required to inhibit 50% of radicals.

### High-performance liquid chromatography analysis

To determine the amount of phenolic and flavonoid compounds, High-Performance Liquid Chromatography (HPLC) analysis was performed on selected plant samples (including control genotypes and genotypes selected based on a cluster analysis). Three grams of dried leaves were extracted by methanol (HPLC-grade, Merck). The extracts were evaporated under reduced pressure to dryness at room temperature. HPLC analysis was performed according to the method of Salami et al.^61^ with some modifications. The extract was filtered and 50 µl of the extract was injected for HPLC analysis. The HPLC analysis was done on an Agilent 1090 and the chromatogram was set at 270 and 330 nm. A 250 mm × 4.6 mm, 5 µm, symmetry C18 column (Waters Corp., Milford, MA, USA) with sentry guard column was utilized at a flow rate of 1 mL/min. The column temperature was set at 25 °C. Solvents A (0.1% formic acid in water) and B (0.1% formic acid in acetonitrile) were used as the mobile phase. The gradient graph was changed from 10% to 26% B (v/v) at 40 min, 65% B at 70 min, and 100% B at 75 min. Phenolic standards used in this study (gallic acid, hydroxybenzoic acid, vanillic acid, syringic acid, ellagic acid, *p*-coumaric acid, rutin, and ferulic acid) were dissolved in methanol. The phenolic compounds were identified by comparison of their retention times with those of pure standards and were expressed as mg per 100 g of dry weight.

### Statistical analysis

In collection of data on plant material, we complied with our relevant institutional and national standards, and also with international guidelines and legislation complied with the IUCN Policy Statement on Research Involving Species at Risk of Extinction and the Convention on the Trade in Endangered Species of Wild Fauna and Flora. Analysis of variance (ANOVA) for traits related to yield components was performed using SAS statistical software (SAS Institute, 1999) according to the augmented design. Cluster analysis (Ward’s method) was conducted using Statgraphics software (Ver 18).

### Supplementary Information

Supplementary Tables.

## Data Availability

All data generated and analyzed during this study are included in this paper. The raw datasets generated during the current study are also available from the corresponding author on a reasonable request.

## References

[1] Morris JB, Wang ML, Morse SA, Kole C (2011). Wild crop relatives: Genomic and breeding resources. Cereals.

[2] Rana M, Dhamija H, Prashar B, Sharma S (2012). *Ricinus communis* L.—A review. Int. J. Pharmtech. Res..

[3] Jena J, Gupta AK (2012). *Ricinus communis* Linn: A phytopharmacological review. Int. J. Pharm. Pharm. Sci..

[4] Singh R, Geetanjali G (2015). Phytochemical and pharmacological investigations of *Ricinus communis* Linn. Alger. J. Nat. Prod..

[5] Abdul W (2018). Therapeutic role of *Ricinus communis* L. and its bioactive compounds in disease prevention and treatment. Asian Pac. J. Trop. Med..

[6] Chouhan HS, Swarnakar G, Jogpal B (2021). Medicinal properties of *Ricinus communis*: A review. Int. J. Pharm. Sci..

[7] Patel VR, Dumancas GG, Viswanath LCK, Maples R, Subong BJJ (2016). Castor oil: Properties, uses, and optimization of processing parameters in commercial production. Lipid Insights.

[8] Bajay MM (2011). Development of a novel set of microsatellite markers for Castor bean, *Ricinus communis* (Euphorbiaceae). Am. J. Bot..

[9] Patel MP, Parmar DJ, Kumar S (2018). Genetic variability, character association and genetic divergence studies in castor (*Ricinus communis* L.). Ann. Agrar. Sci..

[10] de Oliveira IJ, Zanotto MD, Krieger M, Vencovsky R (2012). Inbreeding depression in castor bean (*Ricinus communis* L.) progenies. Crop Breed. Appl. Biotechnol..

[11] Brukhin V (2017). Molecular and genetic regulation of apomixis. Russ. J. Genet..

[12] Li XQ, Donnelly DJ, Jensen TG (2015). Somatic genome manipulation: Advances, methods, and applications. Somat. Genome Manip. Adv. Methods Appl..

[13] Darrigues, A., Daub, J., McCord, K., Rasmussen, C. & Rouse, J. Genetic analysis of apomixis. http://faculty.agron.iastate.edu/madan/Genetics.pdf (2003).

[14] Hand ML, Koltunow AMG (2014). The genetic control of apomixis: Asexual seed formation. Genetics.

[15] Bashaw EC (1980). Apomixis and its application in crop improvement. Hybrid. Crop Plants.

[16] Kandemir N, Saygili I (2015). Apomixis: New horizons in plant breeding. Turkish J. Agric. For..

[17] Bicknell RA (1997). Isolation of a diploid, apomictic plant of *Hieracium aurantiacum*. Sex. Plant Reprod..

[18] Rodriguez-Leal D, Vielle-Calzada JP (2012). Regulation of apomixis: Learning from sexual experience. Curr. Opin. Plant Biol..

[19] Garcia-Aguilar M, Michaud C, Leblanc O, Grimanelli D (2010). Inactivation of a DNA methylation pathway in maize reproductive organs results in apomixis-like phenotypes. The Plant Cell..

[20] Koltunow AM, Grossniklaus U (2003). Apomixis: A developmental perspective. Annu. Rev. Plant Biol..

[21] Pellino M, Hojsgaard D, Hörandl E, Sharbel TF (2020). Chasing the apomictic factors in the *Ranunculus auricomus* complex: Exploring gene expression patterns in microdissected sexual and apomictic ovules. Genes.

[22] Leblanc O, Grimanelli D, Gonzalez de Leon D, Savidan Y (1995). Detection of the apomictic mode of reproduction in maize-*Tripsacum* hybrids using maize RFLP markers. Theor. Appl. Genet..

[23] Bicknell RA, Borst NK (1996). Isolation of reduced genotypes of *Hieracium pilosella* using anther culture. Plant Cell Tissue Organ Cult..

[24] Carneiro, V. T. C., Dusi D. M. A. & Ortiz J. P. A. Apomixis: Occurrence, applications and improvements. *Floric Ornam. Plant Biotechnol. Adv. Top.* 564–571 (2006).

[25] Savidan Y (1981). Genetics and Utilization of Apomixis for the Improvement of Guinea Grass (Panicum maximum Jacq.).

[26] Bicknell RA, Borst NK, Koltunow AM (2000). Monogenic inheritance of apomixis in two *Hieracium* species with distinct developmental mechanisms. Heredity.

[27] Hanna W, Roche D, Ozias-Akins P (1998). Apomixis in crop improvement: traditional and molecular approaches. Advances in Hybrid Rice Technology: Proceeding of the 3rd International Symposium on Hybrid Rice.

[28] Hand ML, Koltunow AMG (2014). The genetic control of apomixis: Asexual seed formation. Genetics.

[29] Ozias-Akins P, Van Dijk PJ (2007). Mendelian genetics of apomixis in plants. Annu. Rev. Genet..

[30] Matzk F, Prodanovic S, Bäumlein H, Schubert I (2005). The inheritance of apomixis in *Poa pratensis* confirms a five locus model with differences in gene expressivity and penetrance. Plant Cell..

[31] Susmita C, Kumar SPJ, Chintagunta AD, Agarwal DK (2022). Apomixis: A foresight from genetic mechanisms to molecular perspectives. Bot. Rev..

[32] Nagarajan S, Viswanathan P, Venkatachalam SR, Manickam S, Ganapathi N (2019). Genetic divergence analysis of castor (*Ricinus communis* L.). Electron. J. Plant Breed..

[33] Sadaiah K (2021). Genetic parameters, diversity and character association studies in germplasm lines of castor (*Ricinus communis* L.). Electron. J. Plant Breed..

[34] Wafa G, Amadou D, Larbi KM, Héla EFO (2016). Larvicidal activity, phytochemical composition, and antioxidant properties of different parts of five populations of
* Ricinus communis
* L.. Ind. Crops Prod..

[35] Vasco-Leal JF (2021). Valorization of Mexican *Ricinus communis* L. leaves as a source of minerals and antioxidant compounds. Waste Biomass Valoriz..

[36] Koutroubas SD, Papakosta DK, Doitsinis A (1999). Adaptation and yielding ability of castor plant (*Ricinus communis* L.) genotypes in a Mediterranean climate. Eur. J. Agron..

[37] Perdomo FA, Acosta-Osorio AA, Herrera G, Vasco-Leal JF (2013). Physicochemical characterization of seven Mexican *Ricinus communis* L. seeds & oil contents. Biomass Bioenergy.

[38] Arif M (2015). Estimating spatial population structure through quantification of oil content and phenotypic diversity in Pakistani castor bean (*Ricinus communis* L.) germplasm. Sci. Technol. Dev..

[39] Nietsche S, Vendrame WA, Crane JH, Pereira MCT (2014). Assessment of reproductive characteristics of *Jatropha curcas* L. in south Florida. GCB Bioenergy.

[40] Francis G, John O, Piergiorgio S, Mulpuri S (2020). Apomixis as a tool for development of high yielding clones and selections in *Jatropha curcas* L.. Genet. Resour. Crop Evol..

[41] Lin JT (2009). Ratios of regioisomers of triacylglycerols containing dihydroxy fatty acids in castor oil by mass spectrometry. J. Am. Oil Chem. Soc..

[42] Lin JT, Chen GQ (2012). Ratios of regioisomers of minor acylglycerols less polar than triricinolein in castor oil estimated by mass spectrometry. J. Am. Oil Chem. Soc..

[43] Hernandez B (2013). Extraction and characterization of *Oecopetalum mexicanum* seed oil. Ind. Crops Prod..

[44] Singh PP, Ambika, Chauhan SMS (2009). Activity guided isolation of antioxidants from the leaves of *Ricinus communis* L.. Food Chem..

[45] Nantitanon W, Yotsawimonwat S, Okonogi S (2010). Factors influencing antioxidant activities and total phenolic content of guava leaf extract. LWT Food Sci. Technol..

[46] Kelley AM, Johnson PG, Waldron BL, Peel MD (2009). A survey of apomixis and ploidy levels among *Poa* l. (Poaceae) using flow cytometry. Crop Sci..

[47] Naumova TN, Hayward MD, Wagenvoort M (1999). Apomixis and sexuality in diploid and tetraploid accessions of *Brachiaria decumbens*. Sex. Plant Reprod..

[48] Singh AP, Mehta DR, Desale CS (2013). Heterosis and inbreeding depression for seed yield and its component traits in castor (*Ricinus communis* L.). Electron. J. Plant Breed..

[49] Patel DK, Patel DA, Patel JR, Patel KV, Parmar DJ (2018). Heterosis and inbreeding depression for seed yield and its contributing characters in castor (*Ricinus communis* L.). J. Pharm. Innov..

[50] Delvadiya IR, Madariya RB, Ginoya AV, Patel JR (2021). Studies on the genetic basis of heterosis and inbreeding depression for seed yield and its component traits in castor (*Ricinus communis* L.). J. Pharm. Innov..

[51] Asker S, Frost S (1970). Chromatographic studies of phenolic compounds in apomictic *Potentilla* L.. Hereditas..

[52] Tateoka T, Tateoka TN (1981). Attributes of *Calamagrostis langsdorflii*, C. sachalinensis and their intermediates in shikoku. Bot. Mag..

[53] Patel K, Patel DK, Watson RR, Preedy VR (2019). The beneficial role of rutin, a naturally occurring flavonoid in health promotion and disease prevention: A systematic review and update. Bioactive Food as Dietary Interventions for Arthritis and Related Inflammatory Diseases.

[54] Schmidt A (2020). Controlling apomixis: Shared features and distinct characteristics of gene regulation. Genes.

[55] Federer WT (1956). Augmented (or hoonuiaku) designs Hawaiian Planters Record. Honolulu.

[56] Firl N, Kienberger H, Hauser T, Rychlik M (2013). Determination of the fatty acid profile of neutral lipids, free fatty acids and phospholipids in human plasma. Clin. Chem. Lab. Med..

[57] Lichtenthaler HK, Wellburn AR (1983). Determinations of total carotenoids and chlorophylls a and b of leaf extracts in different solvents. Biochem. Soc. Trans..

[58] Singleton VL, Rossi JA (1965). Colorimetry of total phenolics with phosphomolybdic-phosphotungstic acid reagents. Am. J. Enol. Vitic..

[59] Marinova D, Ribarova F, Atanassova M (2005). Total phenolics and total flavonoids in bulgarian fruits and vegetables. J. Chem. Technol. Metall..

[60] Brand-Williams W, Cuvelier ME, Berset C (1995). Use of a free radical method to evaluate antioxidant activity. LWT Food Sci. Technol..

[61] Salami M, Rahimmalek M, Ehtemam MH (2016). Inhibitory effect of different fennel (*Foeniculum vulgare*) samples and their phenolic compounds on formation of advanced glycation products and comparison of antimicrobial and antioxidant activities. Food Chem..

