# Supplementary material for: The relation between apomictic seed production and morpho-physiological characteristics in a world collection of castor bean (Ricinus communis L.)
Source: Sci Rep. 2024 Feb 29;14:5013. doi: 10.1038/s41598-024-53700-1 (PMC10904805; doi:10.1038/s41598-024-53700-1)
Supplement: Supplementary file 1 — Supplementary Tables. [file 41598_2024_53700_MOESM1_ESM.docx]

Table S1. Mean comparisons for yield components, agronomic and phytochemical traits in 33 genotypes of castor bean (two modes of reproduction, apomixis and open-pollination) in 2020

| Trait  **Gen** | No. seeds/raceme | | 100 seed weight (g) | | Seed weight/raceme (g) | | | Seed yield/plant (g) | | Oil percentage | | Apomixis ability (%) |
| --- | --- | --- | --- | --- | --- | --- | --- | --- | --- | --- | --- | --- |
|  | Apo | Open | Apo | Open | Apo | Open | Apo | | Open | Apo | Open |  |
| BEN | 38 | 106 | 27.40 | 27.18 | 10.36 | 28.92 | 890.80 | | 2486.69 | 47.92 | 47.50 | 35.85 |
| MEX | 22 | 73 | 23.56 | 23.14 | 5.20 | 16.85 | 348.09 | | 1128.88 | 43.96 | 44.58 | 30.14 |
| ARG1 | 33 | 57 | 27.91 | 24.84 | 9.08 | 14.19 | 790.24 | | 1234.16 | 43.13 | 45.00 | 57.89 |
| JAM | 38 | 59 | 25.33 | 23.01 | 9.57 | 13.66 | 660.57 | | 942.30 | 44.17 | 45.21 | 64.41 |
| ZAF | 35 | 73 | 30.61 | 24.96 | 10.61 | 18.17 | 1813.41 | | 3106.70 | 47.29 | 46.67 | 47.95 |
| ECU | 41 | 65 | 37.20 | 31.57 | 15.23 | 20.51 | 1233.91 | | 1661.06 | 46.46 | 47.92 | 63.08 |
| AFG | 29 | 88 | 28.66 | 27.27 | 8.43 | 24.13 | 1213.25 | | 3474.69 | **51.25✓** | **50.42✓** | 32.95 |
| IND1 | 12 | **18** | 28.64 | 26.51 | 3.31 | **4.75** | 343.994 | | 494.01 | **37.71** | 41.46 | 66.67 |
| CUB1 | 12 | 39 | 34.33 | 29.47 | 3.97 | 11.55 | 440.06 | | 1282.12 | 43.33 | 43.13 | 30.77 |
| MDG | 39 | 82 | 21.91 | 21.08 | 8.51 | 17.23 | 510.77 | | 1033.87 | 46.46 | 45.63 | 47.56 |
| TUR | 23 | 37 | 29.74 | 25.62 | 6.87 | 9.47 | 1573.26 | | 2168.81 | 42.29 | 45.63 | 62.16 |
| USA1 | 28 | 77 | 34.09 | 28.54 | 9.66 | 22.05 | 328.57 | | 749.85 | 42.92 | 45.83 | 36.36 |
| IL | 55 | 121 | **12.76** | **11.70** | 6.97 | 14.16 | 815.34 | | 1656.18 | 42.50 | **41.04** | 45.45 |
| ARG2 | 18 | 69 | 43.00 | 39.67 | 7.68 | 27.55 | **176.61** | | 633.56 | 42.08 | 47.29 | **20.09** |
| IND3 | 25 | 43 | 31.34 | 26.26 | 7.90 | 11.18 | 300.06 | | 424.76 | 46.25 | 45.21 | 58.14 |
| USA2 | 49 | 72 | 27.08 | 29.12 | 13.36 | 20.87 | 1269.61 | | 1982.67 | 44.58 | 46.04 | 68.06 |
| ARG3 | 54 | 72 | 16.23 | 26.99 | 8.69 | 19.35 | 530.29 | | 1180.05 | 43.54 | 45.83 | 75.00 |
| IND2 | **6** | **18** | 45.08 | 31.21 | **2.84** | 5.59 | 204.49 | | **402.65** | 44.17 | 45.42 | 33.33 |
| MAR | 33 | 50 | 39.79 | 30.93 | 12.95 | 15.59 | 1165.53 | | 1403.06 | 45.21 | 45.79 | 66.00 |
| COG | 26 | 48 | 28.99 | 24.50 | 7.61 | 11.80 | 905.47 | | 1404.00 | 45.83 | 41.25 | 54.17 |
| IRN | 77 | 152 | 19.02 | 18.79 | 14.58 | 28.62 | **2813.98✓** | | **5523.60✓** | 41.67 | 47.08 | 50.66 |
| BRA1 | 63 | 73 | 33.14 | 33.92 | 20.88 | 24.69 | 1148.14 | | 1357.98 | 47.18 | 46.87 | **86.30✓** |
| CUB2 | 35 | 99 | 25.76 | 24.32 | 8.93 | 23.97 | 714.08 | | 1917.43 | 43.13 | 42.29 | 35.35 |
| EGY | 20 | 45 | 23.45 | 23.32 | 4.68 | 10.45 | 388.37 | | 866.96 | 47.50 | 44.79 | 44.44 |
| BRA2 | 77 | 148 | 23.56 | 21.77 | 18.06 | 32.18 | 1209.88 | | 2155.91 | 48.13 | 50.00 | 52.03 |
| PRT | 42 | 58 | 31.13 | 33.88 | 13.08 | 19.73 | 784.50 | | 1183.89 | 45.83 | 44.59 | 72.41 |
| BRA3 | 46 | 104 | 22.82 | 21.45 | 10.54 | 22.34 | 200.30 | | 424.44 | 43.75 | 42.50 | 44.23 |
| RUS | 18 | 41 | 34.18 | 33.59 | 6.10 | 13.92 | 634.47 | | 1447.64 | 45.42 | 43.75 | 43.90 |
| SYR | 76 | 125 | 19.86 | 19.73 | 15.02 | 24.75 | 1936.93 | | 3192.37 | 50.83 | 44.79 | 60.80 |
| PAK | 29 | 53 | 34.04 | 31.01 | 10.01 | 16.32 | 690.52 | | 1126.22 | 46.46 | 45.83 | 54.72 |
| THR | 44 | 119 | **47.48✓** | **43.37✓** | 21.01 | **51.41✓** | 264.48 | | 647.40 | 48.96 | 45.83 | 36.97 |
| ISF | 104 | 178 | 20.68 | 18.31 | **25.31✓** | 32.52 | 1769.65 | | 2678.63 | 47.29 | 46.88 | 58.43 |
| Yazd | **114✓** | **182✓** | 22.14 | 19.22 | 21.48 | 34.90 | 1426.81 | | 1967.79 | 50.63 | 50.00 | 62.64 |
| LSD | 31.92 | 11.21 | 9.06 | 9.17 | 1.56 | 1.87 | 188.93 | | 289.76 | 0.25 | 0.12 | 30.29 |

Table S1 (continued)

| Trait  **Gen** | **No. racemes/plant** | | **Height (cm)** | **Diameter (cm)** | **Wet weight (g)** | **Dry weight (g)** | **Chl *a*** | **Chl *b*** | **Carotenoid** | **Phenol** | **Flavonoid** | **IC_50_** |
| --- | --- | --- | --- | --- | --- | --- | --- | --- | --- | --- | --- | --- |
| BEN | 86 | 198.00 | | 4.91 | 2165.91 | 892.15 | 1.22 | 0.37 | 0.31 | 337.45 | 162.62 | 100.56 |
| MEX | 67 | 137.50 | | 3.74 | 828.34 | **388.34** | 1.61 | **0.47✓** | 0.37 | 272.60 | 177.42 | 117.36 |
| ARG1 | 87 | 182.88 | | 4.42 | 1865.63 | 730.41 | 0.56 | **0.15** | **0.15** | 408.40 | 188.19 | 80.07 |
| JAM | 69 | 184.07 | | 4.85 | 1817.25 | 698.52 | 1.36 | 0.35 | 0.36 | 303.12 | 181.46 | 101.05 |
| ZAF | 171 | 197.27 | | 4.80 | 2514.67 | 1061.54 | 0.81 | 0.23 | 0.26 | 362.63 | 156.78 | 87.30 |
| ECU | 81 | 198.00 | | 4.85 | 2289.11 | 830.57 | 0.88 | 0.32 | 0.25 | 267.64 | 188.64 | 128.84 |
| AFG | 144 | 218.90 | | 3.52 | 1436.62 | 651.23 | **1.63✓** | 0.44 | 0.38 | 251.24 | 177.87 | 143.50 |
| IND1 | 104 | 162.25 | | 4.99 | 2106.54 | 811.82 | 1.38 | 0.41 | 0.32 | 332.11 | 185.05 | 100.53 |
| CUB1 | 111 | 189.75 | | 3.54 | 3054.74 | 1078.10 | 1.22 | 0.35 | 0.32 | 351.18 | 206.14 | 95.07 |
| MDG | 60 | **254.47✓** | | 4.33 | 1716.02 | 728.24 | 1.54 | 0.40 | **0.39✓** | 281.76 | 170.69 | 109.39 |
| TUR | **229✓** | 194.33 | | 4.54 | 1729.26 | 812.97 | 1.40 | 0.40 | 0.30 | 242.46 | 133.90 | 145.64 |
| USA1 | 34 | **113.30** | | **3.00** | **638.01** | 288.29 | 0.98 | 0.25 | 0.24 | 339.74 | 144.22 | 110.63 |
| IL | 117 | 210.10 | | 5.49 | 2313.34 | 1042.81 | 1.45 | 0.37 | 0.37 | 469.82 | 181.46 | 78.67 |
| ARG2 | 23 | 188.83 | | 5.41 | 2085.63 | 715.24 | 0.95 | 0.31 | 0.26 | 273.74 | 185.95 | 118.69 |
| IND3 | 38 | 235.40 | | 5.45 | **3102.11✓** | **1217.41✓** | 1.38 | 0.38 | 0.34 | 252.00 | 218.71 | 139.43 |
| USA2 | 95 | 170.50 | | 3.57 | 1912.94 | 750.21 | 1.10 | 0.33 | 0.31 | 445.41 | 175.63 | 69.07 |
| ARG3 | 61 | 205.98 | | 4.66 | 1648.97 | 684.25 | 0.99 | 0.29 | 0.27 | 396.58 | 198.06 | 78.68 |
| IND2 | 72 | 210.93 | | 4.74 | 2176.92 | 833.88 | 1.23 | 0.29 | 0.34 | 277.94 | 156.33 | 111.36 |
| MAR | 90 | 194.70 | | 5.56 | 1835.94 | 746.95 | 1.29 | 0.35 | 0.29 | **176.09** | **120.44** | **214.72✓** |
| COG | 119 | 223.58 | | 4.39 | 1822.71 | 707.32 | 1.44 | 0.40 | 0.37 | 297.78 | 156.33 | 108.45 |
| IRN | 193 | 235.40 | | 4.32 | 2755.52 | 1164.89 | 1.20 | 0.37 | 0.33 | 509.49 | 194.47 | 63.32 |
| BRA1 | 55 | 171.60 | | 4.29 | 1857.96 | 737.45 | 1.10 | 0.28 | 0.31 | 242.46 | 166.65 | 146.25 |
| CUB2 | 80 | 185.17 | | 4.65 | 2085.64 | 872.34 | 1.31 | 0.36 | 0.34 | 289.77 | **220.50✓** | 107.02 |
| EGY | 83 | 149.05 | | 4.20 | 1358.57 | 624.87 | 1.16 | 0.31 | 0.29 | 285.19 | 167.55 | 110.54 |
| BRA2 | 67 | 221.10 | | 5.27 | 2423.35 | 1004.32 | **0.53** | 0.15 | **0.15** | **589.22✓** | 213.77 | **55.65** |
| PRT | 60 | 224.13 | | 4.44 | 1479.53 | 599.54 | 1.29 | 0.40 | 0.32 | 241.70 | 141.97 | 147.68 |
| BRA3 | 19 | 204.33 | | 5.26 | 2008.63 | 667.71 | 1.38 | 0.41 | 0.32 | 282.14 | 159.03 | 110.32 |
| RUS | 104 | 163.63 | | 3.92 | 1383.36 | 537.93 | 0.98 | 0.28 | 0.27 | 247.42 | 135.69 | 140.98 |
| SYR | 129 | 205.98 | | 3.62 | 1783.18 | 772.21 | 1.11 | 0.37 | 0.30 | 325.24 | 176.53 | 105.78 |
| PAK | 69 | 232.10 | | **5.74✓** | 2875.42 | 1035.11 | 1.24 | 0.33 | 0.32 | 287.86 | 144.22 | 104.06 |
| THR | **13** | 218.85 | | 5.28 | 2113.02 | 710.25 | 1.28 | 0.38 | 0.32 | 291.83 | 157.05 | 134.64 |
| ISF | 82 | 202.88 | | 4.83 | 2496.48 | 982.32 | 1.29 | 0.38 | 0.33 | 323.56 | 166.74 | 102.29 |
| Yazd | 56 | 202.05 | | 4.18 | 1672.17 | 671.14 | 1.46 | 0.46 | 0.36 | 289.69 | 161.27 | 145.76 |
| LSD | 10.56 | 7.88 | | 0.17 | 0.34 | 0.11 | 0.03 | 0.02 | 0.02 | 19.98 | 13.35 | 0.08 |

**✓**  indicates the highest values recorded and bolded numbers show the lowest ones.

Table S2. Mean comparisons for yield components, agronomic and phytochemical traits in 33 genotypes of castor bean (two modes of reproduction, apomixis and open-pollination) in 2021

| Trait  **Gen** | No. seeds/raceme | | 100 seed weight (g) | | Seed weight/raceme(g) | | | Seed yield/plant (g) | | Oil percentage | | Apomixis ability (%) |
| --- | --- | --- | --- | --- | --- | --- | --- | --- | --- | --- | --- | --- |
|  | Apo | Open | Apo | Open | Apo | Open | Apo | | Open | Apo | Open |  |
| BEN | 36 | 95 | 31.70 | 26.07 | 9.87 | 25.82 | 808.93 | | 2116.99 | 46.94 | 46.53 | 37.89 |
| MEX | 21 | 65 | 23.83 | 22.97 | 4.95 | 15.04 | 316.67 | | 962.82 | 43.06 | 43.67 | 32.32 |
| ARG1 | 31 | 51 | 32.17 | 26.31 | 8.65 | 12.67 | 718.03 | | 1051.28 | 42.25 | 44.08 | 60.78 |
| JAM | 36 | 53 | 25.54 | 22.94 | 9.12 | 12.19 | 601.79 | | 804.74 | 43.27 | 44.29 | 67.92 |
| ZAF | 33 | 65 | 27.60 | 25.48 | 10.10 | 16.22 | 1646.30 | | 2644.02 | 46.33 | 45.71 | 50.77 |
| ECU | 39 | 58 | 36.40 | 29.99 | 14.51 | 18.31 | 1117.12 | | 1409.87 | 45.51 | 46.94 | 67.24 |
| AFG | 28 | 79 | 28.15 | 27.39 | 8.02 | 21.54 | 1099.29 | | 2951.53 | 49.80 | 48.98 | 35.44 |
| IND1 | 11 | **16** | 28.22 | **56.38✓** | 3.15 | **4.24** | 311.85 | | 419.86 | **36.94** | 40.61 | 68.75 |
| CUB1 | 11 | 35 | 36.00 | 26.32 | 3.78 | 10.31 | 400.26 | | 1093.18 | 42.45 | 42.25 | 31.43 |
| MDG | 37 | 73 | 22.68 | 20.76 | 8.11 | 15.39 | 462.10 | | 876.95 | 45.51 | 44.69 | 50.68 |
| TUR | 22 | 33 | 29.36 | 29.15 | 6.54 | 8.46 | 1426.37 | | 1843.41 | 41.43 | 44.69 | 66.67 |
| USA1 | 27 | 69 | 28.39 | 28.88 | 9.20 | 19.69 | 294.53 | | 630.11 | 42.04 | 44.90 | 39.13 |
| IL | 52 | 108 | **12.43** | **11.47** | 6.64 | 12.64 | 736.71 | | 1402.93 | 41.63 | **40.20** | 48.15 |
| ARG2 | 17 | 62 | 40.89 | 31.72 | 7.31 | 24.60 | **160.89** | | 541.08 | 41.23 | 46.33 | **27.42** |
| IND3 | 24 | 38 | 28.83 | 26.32 | 7.52 | 9.98 | 270.72 | | 359.28 | 45.31 | 44.29 | 63.16 |
| USA2 | 47 | 64 | 26.81 | 29.26 | 12.73 | 18.63 | 1145.52 | | 1677.06 | 43.67 | 45.10 | 73.44 |
| ARG3 | 51 | 64 | 29.06 | 25.98 | 14.95 | 16.73 | 867.04 | | 970.11 | 42.65 | 44.90 | 79.69 |
| IND2 | **6** | **16** | 44.79 | 41.50 | **2.71** | 4.99 | 186.65 | | **344.52** | 43.27 | 44.49 | 37.50 |
| MAR | 31 | 45 | 40.04 | 30.17 | 12.33 | 13.92 | 1060.72 | | 1197.03 | 44.29 | 44.86 | 68.89 |
| COG | 25 | 43 | 30.64 | 24.38 | 7.25 | 10.53 | 818.91 | | 1190.34 | 44.90 | 40.41 | 58.14 |
| IRN | 73 | 136 | 17.67 | 18.03 | 13.89 | 25.55 | **2555.02✓** | | **4701.75✓** | 40.82 | 46.12 | 53.68 |
| BRA1 | 60 | 65 | 34.87 | 32.90 | 19.88 | 22.05 | 1033.81 | | 1146.34 | 46.21 | 45.92 | **92.31✓** |
| CUB2 | 33 | 88 | 27.87 | 25.89 | 8.50 | 21.40 | 646.08 | | 1626.40 | 42.25 | 41.43 | 37.50 |
| EGY | 19 | 40 | 23.19 | 22.57 | 4.46 | 9.33 | 352.02 | | 736.75 | 46.53 | 43.88 | 47.50 |
| BRA2 | 73 | 132 | 23.42 | 22.68 | 17.20 | 28.73 | 1100.67 | | 1838.72 | 47.14 | 48.98 | 55.30 |
| PRT | 40 | 52 | 30.53 | 34.90 | 12.45 | 17.62 | 709.76 | | 1004.17 | 44.90 | 43.68 | 76.92 |
| BRA3 | 44 | 93 | 25.85 | 21.49 | 10.04 | 19.95 | 180.72 | | 359.01 | 42.86 | 41.63 | 47.31 |
| RUS | 17 | 37 | 34.70 | 33.53 | 5.81 | 12.43 | 575.19 | | 1230.37 | 44.49 | 42.86 | 45.95 |
| SYR | 72 | 112 | 21.10 | 19.64 | 14.30 | 22.10 | 1758.90 | | 2717.81 | **50.20✓** | 43.88 | 64.29 |
| PAK | 28 | 47 | 33.33 | 29.89 | 9.53 | 14.57 | 629.05 | | 961.79 | 45.51 | 44.90 | 59.57 |
| THR | 42 | 107 | **48.03✓** | 43.54 | 20.01 | **46.10✓** | 247.43 | | 568.43 | 47.96 | 44.90 | 39.25 |
| ISF | 99 | 159 | 20.93 | 18.30 | 20.65 | 29.03 | 1569.26 | | 2204.05 | 46.33 | 45.92 | 62.26 |
| Yazd | **109✓** | **163✓** | 19.95 | 18.43 | **24.11✓** | 31.16 | 1276.39 | | 1650.14 | 49.59 | **49.39✓** | 66.87 |
| LSD | 11.16 | 17.76 | 6.52 | 12.19 | 4.84 | 5.64 | 280.96 | | 325.64 | 0.57 | 0.31 | 31.61 |

Table S2 (continued)

| Trait  **Gen** | **No. racemes/plant** | | **Height (cm)** | **Diameter (cm)** | **Wet weight (g)** | **Dry weight (g)** | **Chl *a*** | **Chl *b*** | **Carotenoid** | **Phenol** | **Flavonoid** | **IC_50_** |
| --- | --- | --- | --- | --- | --- | --- | --- | --- | --- | --- | --- | --- |
| BEN | 82 | 180.00 | | 4.50 | 1969.34 | 811.22 | 1.38 | 0.42 | 0.34 | 331.73 | 182.36 | 104.70 |
| MEX | 64 | 125.00 | | 3.43 | 753.25 | 353.85 | 1.16 | 0.31 | 0.33 | 282.14 | 146.46 | 107.84 |
| ARG1 | 83 | 166.25 | | 4.05 | 1696.04 | 664.21 | 1.21 | 0.37 | 0.31 | 239.03 | 159.03 | 150.95 |
| JAM | 66 | 167.33 | | 4.45 | 1652.82 | 635.43 | 1.24 | 0.28 | 0.36 | 186.77 | 114.60 | 201.33 |
| ZAF | 163 | 179.33 | | 4.40 | 2286.31 | 965.84 | 1.05 | 0.32 | 0.29 | 379.79 | 151.85 | 85.22 |
| ECU | 77 | 180.00 | | 4.45 | 2081.54 | 755.24 | 0.92 | 0.26 | 0.28 | 289.77 | 175.63 | 103.25 |
| AFG | 137 | 199.00 | | 3.23 | 1306.75 | 592.78 | 1.33 | 0.40 | 0.31 | 334.78 | 143.32 | 106.90 |
| IND1 | 99 | 147.50 | | 4.58 | 1915.37 | 738.57 | 1.02 | 0.31 | 0.28 | 398.49 | 218.26 | 74.20 |
| CUB1 | 106 | 172.50 | | 3.25 | 2777.27 | 980.75 | 1.19 | 0.32 | 0.30 | 252.00 | 155.89 | 139.76 |
| MDG | 57 | 214.00 | | 3.98 | 1560.34 | 662.12 | 1.41 | 0.38 | 0.32 | 276.41 | 171.59 | 113.41 |
| TUR | **218✓** | 176.67 | | 4.17 | 1572.68 | 739.42 | 1.06 | 0.27 | 0.27 | 404.21 | 154.09 | 75.13 |
| USA1 | 32 | **103.00** | | **2.75** | **580.24** | **262.57** | 0.76 | 0.21 | 0.22 | 317.23 | 137.94 | 112.88 |
| IL | 111 | 191.00 | | 5.03 | 2103.28 | 948.25 | 1.40 | 0.36 | 0.34 | 404.97 | 205.24 | 90.80 |
| ARG2 | 22 | 171.67 | | 4.97 | 1896.52 | 650.41 | 1.41 | 0.40 | 0.33 | 282.14 | 137.94 | 108.88 |
| IND3 | 36 | **231.33✓** | | 5.00 | **2820.28✓** | 1058.11 | 1.27 | 0.36 | 0.32 | 425.19 | 205.24 | 76.18 |
| USA2 | 90 | 155.00 | | 3.28 | 1739.51 | 682.71 | 0.66 | 0.21 | 0.19 | 524.37 | 196.72 | 57.95 |
| ARG3 | 58 | 187.25 | | 4.28 | 1499.28 | 622.24 | 1.25 | 0.32 | 0.29 | 218.43 | 174.28 | 172.71 |
| IND2 | 69 | 191.75 | | 4.35 | 1979.51 | 758.91 | **0.63** | **0.17** | **0.17** | 299.68 | **218.71✓** | 100.74 |
| MAR | 86 | 177.00 | | **5.27✓** | 1669.83 | 679.27 | 1.15 | 0.32 | 0.25 | 208.13 | 140.18 | 183.70 |
| COG | 113 | 203.25 | | 4.03 | 1657.59 | 643.24 | 1.25 | 0.31 | 0.33 | 247.80 | 158.13 | 139.94 |
| IRN | 184 | 214.00 | | 3.97 | 2505.75 | **1106.34✓** | **1.62✓** | **0.46✓** | **0.39✓** | 191.35 | 151.85 | 197.94 |
| BRA1 | 52 | 156.00 | | 3.93 | 1689.34 | 670.15 | 1.26 | 0.42 | 0.30 | 214.62 | 166.65 | 175.47 |
| CUB2 | 76 | 168.33 | | 4.27 | 1896.37 | 793.17 | 1.45 | 0.42 | 0.34 | 317.23 | **135.24** | 107.87 |
| EGY | 79 | 135.50 | | 3.85 | 1235.42 | 568.92 | 1.15 | 0.34 | 0.34 | 452.65 | 190.44 | 77.11 |
| BRA2 | 64 | 201.00 | | 4.83 | 2203.24 | 913.71 | 0.99 | 0.27 | 0.29 | 329.44 | 164.41 | 106.07 |
| PRT | 57 | 203.75 | | 4.08 | 1345.28 | 545.46 | 1.28 | 0.34 | 0.34 | 223.01 | 150.50 | 167.93 |
| BRA3 | 18 | 185.75 | | 4.83 | 1826.57 | 607.21 | 1.37 | 0.42 | 0.38 | 212.71 | 184.60 | 178.28 |
| RUS | 99 | 148.75 | | 3.60 | 1258.48 | 489.72 | 0.75 | 0.21 | 0.21 | 257.34 | 139.28 | 136.26 |
| SYR | 123 | 187.25 | | 3.33 | 1621.34 | 702.81 | 1.31 | 0.39 | 0.33 | **657.89✓** | 165.76 | **47.72** |
| PAK | 66 | 211.00 | | 5.10 | 2614.25 | 941.19 | 1.34 | 0.35 | 0.35 | **180.28** | 156.78 | **208.65✓** |
| THR | **12** | 198.95 | | 4.85 | 1938.07 | 646.41 | 1.24 | 0.37 | 0.31 | 267.95 | 148.26 | 131.17 |
| ISF | 76 | 183.33 | | 4.43 | 2269.80 | 893.21 | 1.15 | 0.36 | 0.31 | 352.48 | 159.39 | 103.92 |
| Yazd | 53 | 183.63 | | 3.84 | 1520.07 | 610.61 | 1.18 | 0.34 | 0.31 | 390.70 | 167.82 | 99.37 |
| LSD | 19.77 | 0.22 | | 0.24 | 17.46 | 0.44 | 0.02 | 0.02 | 0.01 | 24.84 | 10.67 | 0.36 |

**✓**  indicates the highest values recorded and bolded numbers show the lowest ones.
